# Supplementary material for: Novel Driver Strength Index highlights important cancer genes in TCGA PanCanAtlas patients
Source: PeerJ. 2022 Aug 11;10:e13860. doi: 10.7717/peerj.13860 (PMC9375969; doi:10.7717/peerj.13860)
Supplement: Supplemental Information 2 [file peerj-10-13860-s002.zip › COHORTS/genes plots/cohorts/2021_8_16_14_9_distribution_cohorts_males_READ.pdf]

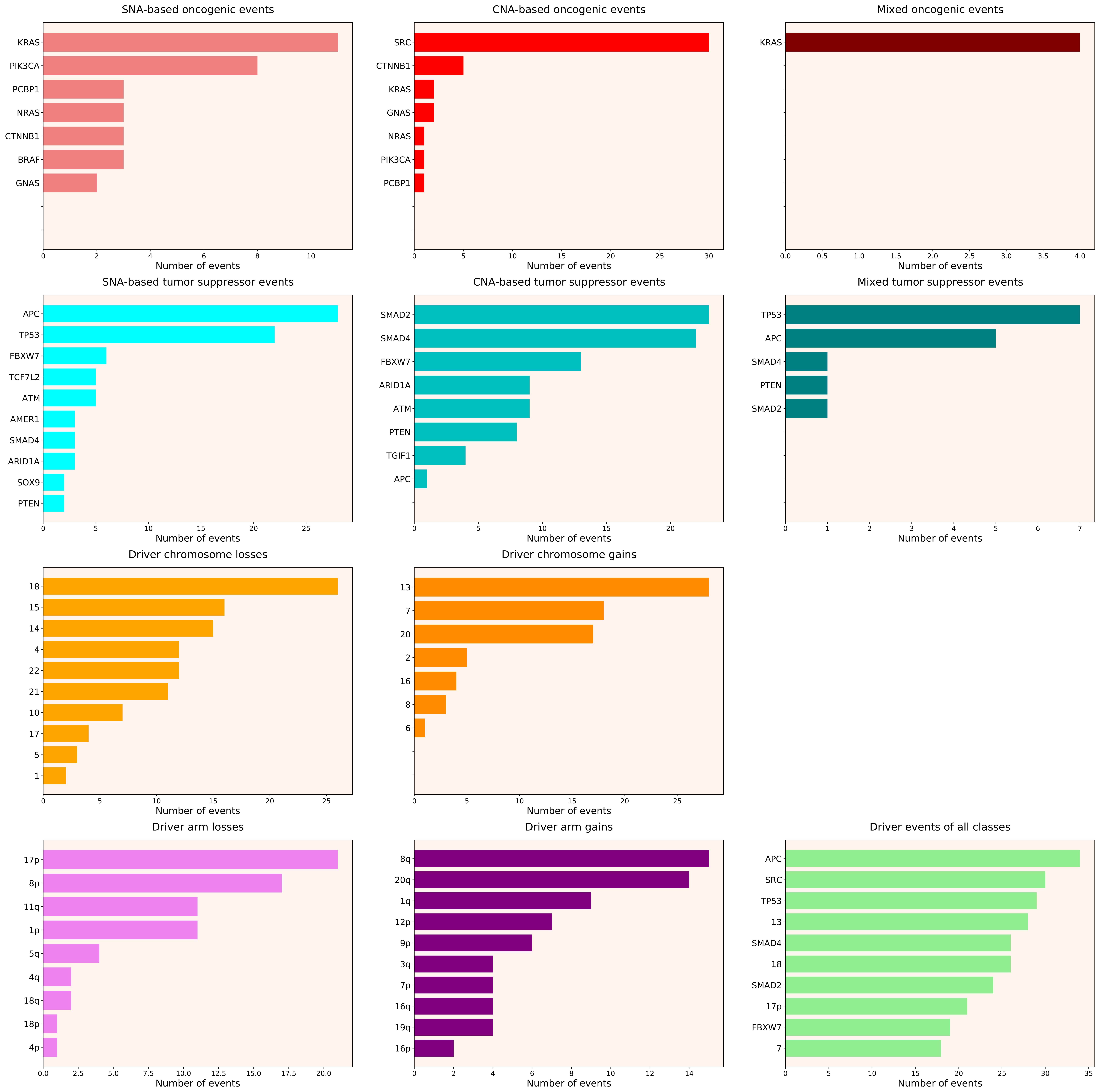
SNA-based oncogenic events
KRAS
PIK3CA
PCBP1
NRAS
CTNNB1
BRAF
GNAS
0 2 4 6 8 10
Number of events
CNA-based oncogenic events
SRC
CTNNB1
KRAS
GNAS
NRAS
PIK3CA
PCBP1
0 5 10 15 20 25 30
Number of events
Mixed oncogenic events
KRAS
0.0 0.5 1.0 1.5 2.0 2.5 3.0 3.5 4.0
Number of events
SNA-based tumor suppressor events
APC
TP53
FBXW7
TCF7L2
ATM
AMER1
SMAD4
ARID1A
SOX9
PTEN
0 5 10 15 20 25
Number of events
CNA-based tumor suppressor events
SMAD2
SMAD4
FBXW7
ARID1A
ATM
PTEN
TGIF1
APC
0 5 10 15 20
Number of events
Mixed tumor suppressor events
TP53
APC
SMAD4
PTEN
SMAD2
0 1 2 3 4 5 6 7
Number of events
Driver chromosome losses
18
15
14
4
22
21
10
17
5
1
0 5 10 15 20 25
Number of events
Driver chromosome gains
13
7
20
2
16
8
6
0 5 10 15 20 25
Number of events
Driver arm losses
17p
8p
11q
1p
5q
4q
18q
18p
4p
0.0 2.5 5.0 7.5 10.0 12.5 15.0 17.5 20.0
Number of events
Driver arm gains
8q
20q
1q
12p
9p
3q
7p
16q
19q
16p
0 2 4 6 8 10 12 14
Number of events
Driver events of all classes
APC
SRC
TP53
13
SMAD4
18
SMAD2
17p
FBXW7
7
0 5 10 15 20 25 30 35
Number of events
